# Albendazole sensitizes cancer cells to ionizing radiation

**DOI:** 10.1186/1748-717X-6-160

**Published:** 2011-11-17

**Authors:** Kirtesh Patel, Nicole A Doudican, Peter B Schiff, Seth J Orlow

**Affiliations:** 1 The Ronald O. Perelman Department of Dermatology, New York University School of Medicine, 550 First Avenue, New York, NY, 10016, USA; 2 Department of Radiation Oncology, New York University School of Medicine, 550 First Avenue, New York, NY, 10016, USA; 3 Department of Cell Biology, New York University School of Medicine, 550 First Avenue, New York, NY, 10016, USA; 4 New York University Cancer Institute, 160 East 34th Street, New York, NY, 10016, USA

**Keywords:** Albendazole, ionizing radiation, DNA damage, microtubules, apoptosis

## Abstract

**Background:**

Brain metastases afflict approximately half of patients with metastatic melanoma (MM) and small cell lung cancer (SCLC) and represent the direct cause of death in 60 to 70% of those affected. Standard of care remains ineffective in both types of cancer with the challenge of overcoming the blood brain barrier (BBB) exacerbating the clinical problem. Our purpose is to determine and characterize the potential of albendazole (ABZ) as a cytotoxic and radiosensitizing agent against MM and SCLC cells.

**Methods:**

Here, ABZ's mechanism of action as a DNA damaging and microtubule disrupting agent is assessed through analysis of histone H2AX phosphorylation and cell cyle progression. The cytotoxicity of ABZ alone and in combination with radiation therapy is determined though clonogenic cell survival assays in a panel of MM and SCLC cell lines. We further establish ABZ's ability to act synergistically as a radio-sensitizer through combination index calculations and apoptotic measurements of poly (ADP-ribose) polymerase (PARP) cleavage.

**Results:**

ABZ induces DNA damage as measured by increased H2AX phosphorylation. ABZ inhibits the growth of MM and SCLC at clinically achievable plasma concentrations. At these concentrations, ABZ arrests MM and SCLC cells in the G2/M phase of the cell cycle after 12 hours of treatment. Exploiting the notion that cells in the G2/M phase are the most sensitive to radiation therapy, we show that treatment of MM and SCLC cells treated with ABZ renders them more sensitive to radiation in a synergistic fashion. Additionally, MM and SCLC cells co-treated with ABZ and radiation exhibit increased apoptosis at 72 hours.

**Conclusions:**

Our study suggests that the orally available antihelminthic ABZ acts as a potent radiosensitizer in MM and SCLC cell lines. Further evaluation of ABZ in combination with radiation as a potential treatment for MM and SCLC brain metastases is warranted.

## Introduction

Melanoma and small cell lung cancer (SCLC) have a high propensity for metastasizing to the brain, accounting for the most common and third most common cause of brain metastasis, respectively [1]. With no currently FDA-approved agents that cross the blood brain barrier (BBB) to target SCLC, conventional therapy for brain metastasis is limited to whole brain radiation therapy. Even though SCLC is radiosensitive, patients receiving prophylactic cranial irradiation after a complete response to chemotherapy still have a 33% 3-year brain metastasis rate and only a 21% 3 year-overall survival rate [2]. While the standard of care for melanoma brain metastases is temozolomide (TMZ) and whole brain radiation therapy (WBRT), this combination therapy does not improve overall survival with this radioresistant tumor [3,4]. The poor prognoses of SCLC and melanoma brain metastases highlight the need for an effective radiosensitizer that can cross the BBB and offer more effective systemic therapy.

Recently, we have shown that mebendazole (MBZ), a marketed benzimidazole (BZ) antihelminthic, is an effective anti-melanoma agent given its ability to disrupt microtubule stability at clinically achievable concentrations, thereby inducing apoptosis [5]. Albendazole (ABZ) is another marketed antihelminthic that is structurally related to MBZ. ABZ, however, has the unique advantage of crossing the BBB, a characteristic that is used to treat parasitic infections of the central nervous system and may be harnessed to potentially target brain metastasis [6]. Although ABZ is structurally similar to MBZ, our data suggests that ABZ also possesses DNA damaging capabilities.

With both metastatic melanoma (MM) and SCLC having a high propensity of brain metastases, we hypothesized that ABZ would be a potent chemotherapeutic and radiosensitizing agent for melanoma and SCLC brain metastases. We show here that at clinically achievable plasma concentrations, ABZ decreases proliferation, which correlates with arrest of the cancer cells in the G2/M phase of the cell cycle. We establish that ABZ radiosensitizes MM and SCLC and that this effect is synergistic. Radiosensitization by ABZ is characterized by enhanced radiation induced apoptosis.

## Materials and methods

### Cell culture

A375 and A2058 metastatic melanoma cells lines were obtained from ATCC (Manassas, VA). H153 and H446 SCLC lines were generously provided by Drs. J. Donnington and H. Sauthoff (New York University School of Medicine, New York, NY), respectively. All cell lines were maintained in Dulbecco's modified Eagle's medium supplemented with 10% fetal bovine serum, 5 units/mL penicillin, and 5% glutamate. All cells were incubated at a humidified atmosphere of 5% CO_2 _at 37°C. ABZ was purchased from Sigma (St Louis, MO).

### Cell Proliferation Assay

All cells were seeded in 96-well plates at a density of 5000 cells per well. After overnight incubation, cells were treated with ABZ at concentrations ranging from 100 nM to 1000 nM. After 12 or 72 hr incubation periods, proliferation was assessed using a tetrazolium dye reduction assay (CellTiter 96 AQueous Non-Radioactive Cell Proliferation Assay; Promega, Madison, WI) [5]. Absorbance was recorded on a microplate reader at 495 nm. Cellular proliferation was expressed as a percentage with vehicle-treated cells set at 100%. Each assay was performed in triplicate with mean values reported.

### Cell Cycle Analysis

A2058 and H153 cells were seeded at 50,000 cells/ml in a 10 cm^2 ^tissue culture dish and incubated overnight. Cells were treated with ABZ at 100 nM and 500 nM. After 6, 12, 18, and 24 hrs, cells and media were collected, washed with 1× PBS, and fixed in 70% ethanol at 4°C. Following incubation in ethanol, cells were washed in phosphate citrate buffer (0.1 M citric acid, 0.2 M Na_2_HPO_4_, pH 7.8), treated with 5 μg Rnase A and incubated in 10 μg propidium iodide solution (Sigma, St Louis, MO). Cell-cycle distribution was determined by flow cytometery of at least 10,000 gated cells using a FACScan flow cytometer. All cell cycle analyses were performed at least twice.

### Clonogenic Survival Assay

Cells were seeded in 6 well plates at a density of 200-400 cells per well. After an 8 hr attachment period, cells were treated with ABZ at 100 nM or 500 nM for 12 hrs. The cells were then unirradiated (control) or irradiated with 6 MV photons (0.300 Gy/min) for total doses ranging from 1 to 8 Gy. Immediately after radiation, media containing ABZ was exchanged for fresh media. Cells were subsequently cultured for approximately 2 weeks, with media changed every third day. Next, colonies were stained with 0.1% crystal violet and counted. The percent plating efficiency and surviving fraction were calculated based on the survival of non ABZ treated, nonirradiated cells treated. Each assay was performed in doublet with the mean reported.

### Calculation of Radiation-ABZ Interactions

The results from the clonogenic assay measurements were analyzed using Calcusyn software (Biosoft, Cambridge, UK). The program calculates the "Combination Index" [7] based upon the methods of Chou and Talalay [8]. CI < 0.90 is indicative of synergistic interactions, CI of 0.90 to 1.0 indicates additive interactions, and CI > 1.0 indicates antagonistic interactions.

### Western Blot Analysis

A2058 and H153 cells were harvested after treatment with 0, 100, or 500 nM ABZ for 12 hrs. Cells were then irradiated at 2 or 10 Gy or left unirradiated as a control. Immediately after radiation, media was changed and cells were incubated for 72 hrs. Cells were then collected, washed in ice cold 1× PBS, and harvested in an extraction buffer (1% Triton X-100, 50 mmol/L Tris, 2 mmol/L EDTA, 150 mmol/L NaCl, pH 7.5) containing protease inhibitor cocktail (Roche Applied Science, Indianapolis, IN) and phosphatase inhibitor cocktail (Sigma, St Louis, MO). The lysates were centrifuged at 14,000 × *g*, 4°C for 20 min in a microcentrifuge. The protein concentrations of supernatants were measured with a protein assay kit (Bio-Rad, Hercules, CA). Proteins were separated by 12% SDS-PAGE and transferred onto polyvinylidene difluoride membranes (Polyscreen, Perkin-Elmer). For apoptotic studies, antibodies against cleaved forms of poly (ADP-ribose) polymerase (PARP) and caspase 9 were obtained from Cell Signaling Technology (Danvers, MA). For pH2AX studies, mouse monoclonal anti-pH2HAX IgG (Santa Cruz Biotechnology, Santa Cruz, CA) was used. For all blots, anti-actin (Sigma, St Louis, MO) was used as a loading control. Immunoreactive bands were visualized using enhanced chemiluminescence detection reagent (Perkin-Elmer, Waltham, MA) and X-OMAT processing.

### Statistics

Unless otherwise noted, experiments were performed in triplicate. All data are presented as the average ± standard error of the mean (SEM). *P*-values were determined by a two-sided Student's *t*-test with unequal variance, with *P *< 0.05 considered significant.

## Results

### Albendazole treatment causes phosphorylation of H2AX

**Figure 1:**
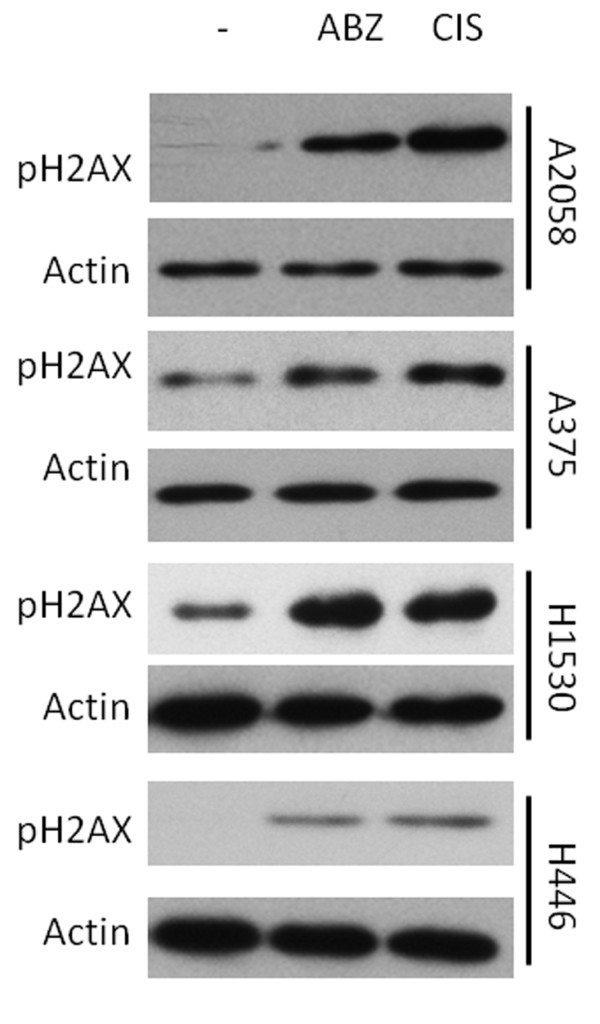
**Albendazole treatment causes phosphorylation of H2AX**. A2058 melanoma cells received no treatment (-) or treatment with 500 nM albendazole (ABZ) or 1 μM cisplatin (CIS) for 9 hr. Extracted proteins analyzed by Western blotting using an antibody against phosphorylated H2AX with actin used as a loading control.

From a screen of 2000 compounds, we identified 4 BZs that were selectively cytotoxic to melanoma cells as compared to normal melanocytes [5]. In order to identify potential mechanisms of action for clinically relevant BZs, the growth inhibitory properties of MBZ and ABZ were examined in the NCI 60 cell screen as part of the Developmental Therapeutics Program at the NCI/NIH. The GI_50 _(concentration needed to achieve 50% growth inhibition) data was then analyzed using COMPARE, an algorithm that can predict possible mechanism(s) of action for compounds based upon patterns of growth inhibitory activity in the NCI 60 cell screen [9]. Based upon this analysis, MBZ shared overlapping activity with antimitotic agents such as rhioxin and paclitaxel (correlation = 0.639 and 0.436, respectively). In contrast, ABZ's spectrum of activity is most similar to that of the DNA damaging agent methyl-cyclonexyl-chloroethyl-nitrosourea (methyl-CCNU; correlation = 0.636). Therefore, we tested the hypothesis that ABZ acts as a DNA damaging agent using phosphorylation of the histone subunit as an indicator of DNA damage. Consistent with the COMPARE results, treatment of MM and SCLC cells with ABZ showed an increase in phosphorylation of a member of the histone H2A family, H2AX (Figure 1). Cisplatin, a direct DNA damaging agent that serves as a positive control, induced similar levels of H2AX phosphorylation after treatment (Figure 1).

### ABZ inhibits growth of MM and SCLC cells *in vitro*

**Figure 2:**
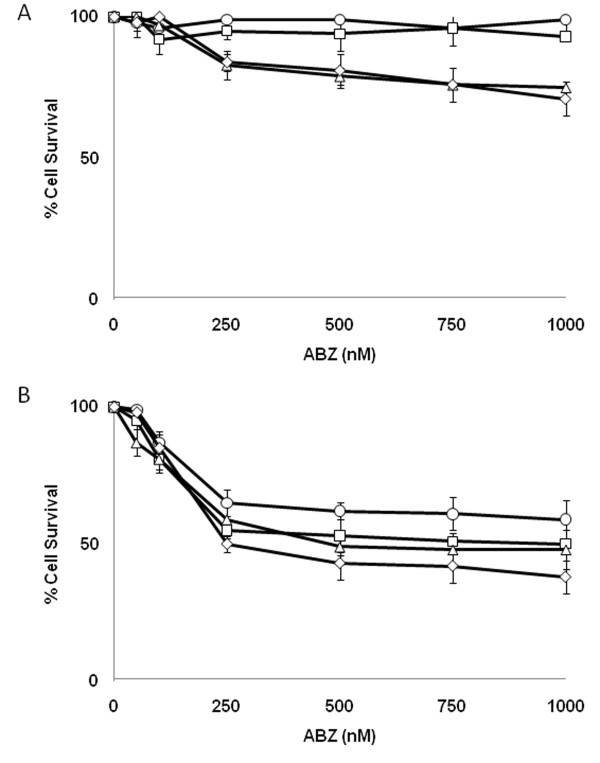
**Albendazole inhibits proliferation of MM and SCLC cells**. A375 (○), A2058 (□), H1530 (◊) and H446 cells (Δ) were treated with increasing concentrations of ABZ for 12 hours (A) or 72 hours [2]. *Points*, mean from a minimum of four independent experiments. *Bars*, SEM.

Given ABZ's ability to cross the BBB along with its joint activity as a DNA damaging agent and microtubule destabilizer, we sought to investigate its potential as a radiosensitizer. Initially, we examined the effects of single agent ABZ on the proliferation of two types of cancer with a high rate of brain metastasis: MM and SCLC. Treatment with clinically achievable concentrations of ABZ inhibited proliferation of both MM (A2058 and A375) and SCLC (H153 and H446) cells. After 72 hr treatment with 500 nM ABZ, a 37.8% inhibition of A375 growth and 46.9% of A2058 growth was observed (Figure 2b). Similarly, treatment with ABZ inhibited SCLC cellular proliferation. H153 and H446 respectively displayed 56.9% and 30.3% growth inhibition after treatment with 500 nM ABZ for 72 hours (Figure 2b). Concentrations of ABZ greater than 500 nM did not result in significant further growth inhibition in the cell lines examined.

### Albendazole induces cell cycle arrest at G_2_/M

Given ABZ's well-characterized effects as a microtubule disrupting agent [10], we wanted to determine if inhibition of cellular proliferation in MM and SCLC cells might result from cell cycle arrest. In MM and SCLC cells, ABZ arrests cells in the G_2_/M phase in a dose-dependent manner when compared to untreated cells (Table 1). Specifically, after 12 hours of ABZ treatment, the percent of A375 melanoma cells in the G2/M phase increased from 4.7 to 65.2 upon treatment with 500 nM ABZ. H153 and H446 SCLC and A2058 MM cells showed a similar trend at 12 hr treatment with 500 nM ABZ (Table 1), thereby indicating that ABZ causes increased MM and SCLC cell accumulation at G_2_/M phase of the cell cycle.

**Table 1:** Albendazole arrests cell in the G2/M phase in a time and dose dependent fashion.

	% G_2_/M cells				
**Time (hr)**	**ABZ (nM)**	**A2058**	**A375**	**H1530**	**H446**

0	0	10.6	4.7	4.3	0.9

12	100	10.0	13.1	3.0	5.2

12	500	29.3	65.2	38.6	47.7

12	1000	32.3	87.1	46.3	71.4

A2058 melanoma cells and H1530 SCLC cells were treated with ABZ concentrations ranging from 100 to 1000 nM for 12 hr, stained with propidium iodide, and analyzed for DNA content by flow cytometry. A total of 10 000 nuclei were analyzed from each sample, with the average of two replicates reported.

### Albendazole radiosensitizes MM and SCLC cells

**Figure 3:**
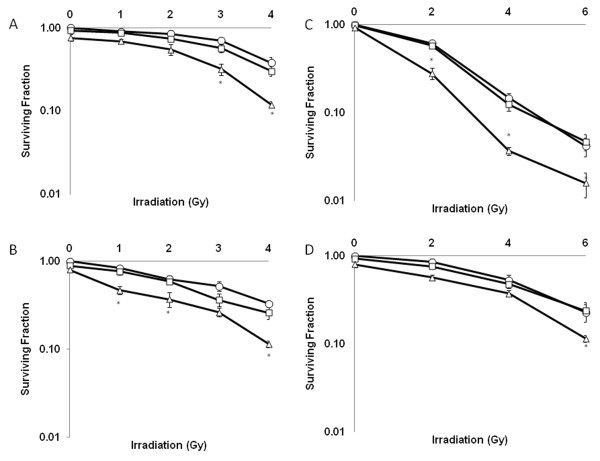
**Treatment with albendazole sensitizes MM and SCLC cells to ionizing radiation**. Radiosensitization by albendazole was assessed on the basis of clonogenic cell survival assays. H446 (A), H1530 [2], A375 (C) and A2058 (D) cells were treated with 0 (О), 100 (□), or 500 nM (Δ) ABZ for 12 hr then irradiated as indicated. Cells were stained with crystal violet after being cultured for approximately 2 weeks. *Points*, average of experiments each plated in duplicate. *Bars*, SEM. *, P < 0.05.

Because ABZ's activity as a microtubule inhibitor is similar to the radiosensitizing paclitaxel [11], we decided to study ABZ's radiosenitizing potential. It was important to consider ABZ's potential as a radiosensitizer at shorter incubation periods given that the side effects in humans are dose and time dependent. While 72 hr of ABZ treatment resulted in growth inhibition of MM and SCLC (Figure 2b), G2/M arrest was accompanied by minimal growth inhibition in cell lines treated with ABZ for 12 hr (Figure 2a). Therefore, we decided to assess if ABZ can radiosensitize at shorter time points. As shown in Figure 3, treatment with 500 nM ABZ promoted radiation-induced clonogenic death in a dose-dependent manner in A2058 and A375 melanoma cells as well as H446 and H1530 SCLC cells. Survival fraction at 2 Gy (SF2), calculated by dividing the number of surviving clones at 2 Gy by the number of surviving clones receiving no radiation or ABZ treatment, for A375 cells was reduced from 61.6% in the irradiated-only controls to 28.1% in ABZ treated and irradiated cells (Table 2). A375 cells treated only with 500 nM ABZ for 12 hrs showed a survival of 93.2% (Table 2; Figure 3). The SF2 for the combination treatment (28.1%) was greater than the added effects of either radiation or ABZ alone (Table 2). A2058 and SCLC cells also demonstrated similar supra-additive responses to ABZ and radiation (Figure 3; Table 2). Moreover, as depicted in Table 3 synergistic effects (CI < 0.90) on all 4 cells colony formation were found when pretreated with 500 nM ABZ followed by irradiating at 2 Gy. Although treatment at 100 nM decreased SF2 and demonstrated an additive effect (CI = 0.90-1.0), the difference with irradiated-only control cells was not statistically significant. Survival enhancement ratios (SER10) were calculated at 10% cell survival by dividing radiation dose of the radiation only survival curve with that of the corresponding ABZ plus radiation curve.

**Table 2:** ABZ induces a lower SF2 (ie supra-additive) than the expected added SF2 (EAS) calculated by adding the effect of radiation alone and ABZ alone.

		% Survival		
**Cell line**	**ABZ (nM)**	**0 Gy**	**2 Gy**	**EAS**

A375	0	100	61.6	

	100	101.3	57.8	

	500	93.2	28.1	53.8

A2058	0	100	85.0	

	100	93.4	76.2	

	500	80.2	56.8	64.8

H1530	0	100	62.8	

	100	89.4	58.9	

	500	80.5	37.1	43.3

H446	0	100	84.7	

	100	92.6	73.3	

	500	75.6	54.5	60.3

**Table 3:** Albendazole acts synergistically with radiation in MM and SCLC cells.

		% Survival		
**Cell line**	**ABZ (nM)**	**CI Values**	**SER**	**Plating Eff**.

A375	100	0.981		

	500	0.701	1.45	43%

A2058	100	0.921		

	500	0.675	1.32	29%

H1530	100	0.89		

	500	0.528	1.28	34%

H446	100	0.906		

	500	0.485	1.20	26%

Combination Index values for ABZ and radiation. Values less than 0.9 indicate a synergistic relationship, values between 0.9 and 1.0 indicate an additive relationship, and values greater than 1.0 indicate an antagonistic relationship.

### Albendazole enhances radiation-induced apoptosis in MM and SCLC cells

**Figure 4:**
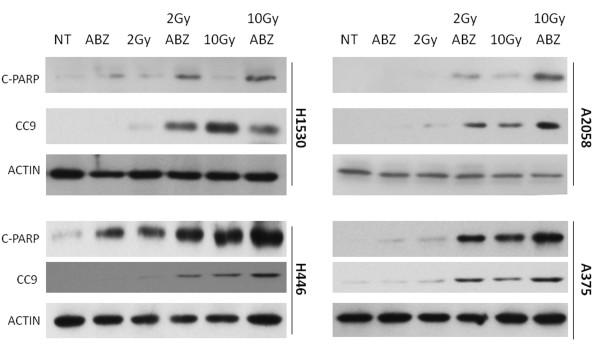
**ABZ treatment potentiates apoptosis in irradiated melanoma and SCLC cells**. A2058 and H1530 cells were treated with 500 nM ABZ, 2 Gy or 10 Gy radiation, or indicted combination. Proteins were analyzed by Western blot hybridization with an antibody against cleaved PARP (C-PARP) or cleaved caspase 9 (CC9) antibody. Actin served as loading control. NT = No treatment.

Having established ABZ as a radiosensitizer, we decided to characterize this effect by determining if pretreatment with ABZ resulted in potentiation of radiation-induced apoptosis as measured by levels of cleaved PARP, a marker of apoptosis. Figures 3a and 3b show that the combination 12 hours of ABZ pretreament followed by irradiation resulted in enhanced PARP cleavage as compared to treatment with either agent alone. Because both MM and SCLC cells demonstrated increased PARP cleavage with the combination treatment compared to the effects of radiation or ABZ alone. As an additional marker of apoptosis, cleavage of caspase 9 was assessed in MM and SCLC cells treated with ABZ and radiation in combination. Consistent with cleavage of PARP, cells receiving combination ABZ and radiation treatment demonstrated enhanced levels of cleaved caspase 9 as compared to treatment with either single agent alone. Cleavage of caspase 9 is indicative of activation of the intrinsic, mitochondrial-mediated apoptotic pathway. In all 4 cell lines examined, the level of cleaved PARP and caspase 9 observed in cells treated with 2 Gy irradiation and ABZ were comparable to levels observed upon treatment with 10 Gy irradiation alone. Altogether, these data suggest that ABZ sensitizes these cells to radiation induced apoptosis (Figure 4).

## Discussion

Here, we demonstrate that ABZ, a BZ that effectively crosses the BBB, is cytotoxic to MM as well as SCLC (Figure 2). We found that this effect correlates with cellular arrest at the G2/M phase of the cell cycle (Table 1). We show ABZ's ability to act synergistically with radiation through combination index analysis (Table 3). ABZ achieves this effect in part by damaging DNA and increasing radiation-induced apoptosis (Figure 1 and 4).

Our study has potential clinical significance. ABZ is being explored as a potent *in vitro *and *in vivo *anti-neoplastic agent [12,13], and here we demonstrate that it has further clinical potential as a radiosensitizer. The radiation dose of 2 Gy used in this study closely approximates the dose per fraction (2 to 3 Gy) used in patients receiving WBRT for metastatic melanoma [14] or prophylactic cranial irradiation for SCLC [3]. Additionally, the concentration of ABZ employed in our studies is readily achievable by oral administration [6]. A dose escalation phase 1 clinical trial of ABZ in 33 patients with a variety of metastatic cancers showed a maximum achievable plasma concentration after oral administration to be 10.25 μM [15]. With other pharmacokinetic studies showing that average cerebrospinal fluid (CSF) concentration is approximately half the serum plasma concentration [6], our *in vitro *concentration of 500 nM is clinically achievable and well tolerated in patients with brain metastases. Furthermore, we observe radiosensitization at concentrations significantly lower than reported maximal CSF concentrations. At these lower concentrations, the incidence of side effects is expected to be reduced.

Like paclitaxel, ABZ is considered a spindle poison [10]. At low clinical concentrations (less than 10% of the maximum plasma achievable concentration in humans), paclitaxel induces G2/M cell cycle arrest [16,17]. With the G2/M phase being the most radiosensitive phase, paclitaxel has been show to sensitize melanoma to radiation, inducing a SER of 1.2 at 40 nM [18]. Similarly, we show here that at low clinical concentrations, ABZ arrests cells in the G2/M phase and radiosensitizes melanoma, inducing a higher SER of 1.39. We hypothesize this difference in SER for comparable concentrations of ABZ and paclitaxel to be due to ABZ's ability to simultaneously induce DNA damage (Figure 1). Previous work has indicated that other BZ's possess DNA damaging capabilities [19,20]. Moreover, additional studies have reported that paclitaxel-resistant cell lines are sensitive to ABZ, suggestive of a mechanism of action in addition to tubulin disruption [21,22]. Indeed, data obtained from COMPARE suggesting that ABZ possesses a spectrum of activity most similar to the DNA damaging agent methyl-CCNU is consistent with this notion. Furthermore, ABZ is also a more suitable clinical agent for radiosensitization because unlike paclitaxel, it can cross the BBB to target melanoma or SCLC brain metastases. In conclusion, ABZ's ability to cross the BBB, radiosensitize cancer cells like melanoma and SCLC which carry a high propensity for metastasizing to the brain warrants further investigations of clinical application.

## Conclusion

The marketed anti-parasitic drug albendazole possesses DNA damaging and microtubule disrupting capabilities. Based upon this unique spectrum of activity as well as its ability to cross the blood brain barrier, we examined albendazole's potential radiosensitization capabilities. At clinically achievable concentrations, albendazole in combination with irradiation causes enhanced apoptotic induction in melanoma and SCLC, which have an increased propensity to metastasize to the brain.

## Competing interests

The authors declare that they have no competing interests.

## Authors' contributions

KP conceived of the study and carried out experiments described herein. ND performed the CI calculations, assisted in experimental design and aided KP in carrying out experiments. KP and ND drafted the manuscript. SJO and PS participated in experimental design and coordination. All authors have read and approved the final manuscript.

## References

[B1] Johnson JD Young B Demographics of brain metastasis Neurosurg Clin N Am 1996 7 3 337 44 8823767

[B2] Auperin A Arriagada R Pignon JP Le Pechoux C Gregor A Stephens RJ Prophylactic cranial irradiation for patients with small-cell lung cancer in complete remission. Prophylactic Cranial Irradiation Overview Collaborative Group N Engl J Med 1999 341 7 476 84 10.1056/NEJM199908123410703 10441603

[B3] Margolin K Atkins B Thompson A Ernstoff S Weber J Flaherty L Temozolomide and whole brain irradiation in melanoma metastatic to the brain: a phase II trial of the Cytokine Working Group J Cancer Res Clin Oncol 2002 128 4 214 8 10.1007/s00432-002-0323-8 11935312 PMC12164456

[B4] Hofmann M Kiecker F Wurm R Schlenger L Budach V Sterry W Temozolomide with or without radiotherapy in melanoma with unresectable brain metastases J Neurooncol 2006 76 1 59 64 10.1007/s11060-005-2914-0 16132502

[B5] Doudican N Rodriguez A Osman I Orlow SJ Mebendazole induces apoptosis via Bcl-2 inactivation in chemoresistant melanoma cells Mol Cancer Res 2008 6 8 1308 15 10.1158/1541-7786.MCR-07-2159 18667591

[B6] Jung H Hurtado M Sanchez M Medina MT Sotelo J Plasma and CSF levels of albendazole and praziquantel in patients with neurocysticercosis Clin Neuropharmacol 1990 13 6 559 64 10.1097/00002826-199012000-00008 2276121

[B7] Canestraro M Galimberti S Savli H Palumbo GA Tibullo D Nagy B Synergistic antiproliferative effect of arsenic trioxide combined with bortezomib in HL60 cell line and primary blasts from patients affected by myeloproliferative disorders Cancer Genet Cytogenet 2010 199 2 110 20 10.1016/j.cancergencyto.2010.02.010 20471514

[B8] Chou TC Talalay P Quantitative analysis of dose-effect relationships: the combined effects of multiple drugs or enzyme inhibitors Adv Enzyme Regul 1984 22 27 55 6382953 10.1016/0065-2571(84)90007-4

[B9] Zaharevitz DW Holbeck SL Bowerman C Svetlik PA COMPARE: a web accessible tool for investigating mechanisms of cell growth inhibition J Mol Graph Model 2002 20 4 297 303 10.1016/S1093-3263(01)00126-7 11858638

[B10] Gupta RS Cross-resistance of nocodazole-resistant mutants of CHO cells toward other microtubule inhibitors: similar mode of action of benzimidazole carbamate derivatives and NSC 181928 and TN-16 Mol Pharmacol 1986 30 2 142 8 3736539

[B11] Lacey E Brady RL Prichard RK Watson TR Comparison of inhibition of polymerisation of mammalian tubulin and helminth ovicidal activity by benzimidazole carbamates Vet Parasitol 1987 23 1-2 105 19 10.1016/0304-4017(87)90029-X 3564338

[B12] Cai ZY Galettis P Lu Y Morris DL Pourgholami MH Pharmacokinetics of albendazole in New Zealand white rabbits: oral versus intraperitoneal administration Anticancer Res 2007 27 1A 417 22 17352262

[B13] Pourgholami MH Woon L Almajd R Akhter J Bowery P Morris DL In vitro and in vivo suppression of growth of hepatocellular carcinoma cells by albendazole Cancer Lett 2001 165 1 43 9 10.1016/S0304-3835(01)00382-2 11248417

[B14] Rades D Heisterkamp C Huttenlocher S Bohlen G Dunst J Haatanen T Dose escalation of whole-brain radiotherapy for brain metastases from melanoma Int J Radiat Oncol Biol Phys 2010 77 2 537 41 10.1016/j.ijrobp.2009.05.001 19733017

[B15] Pourgholami MH Szwajcer M Chin M Liauw W Seef J Galettis P Phase I clinical trial to determine maximum tolerated dose of oral albendazole in patients with advanced cancer Cancer Chemother Pharmacol 2010 65 3 597 605 10.1007/s00280-009-1157-8 19904538

[B16] Sonnichsen DS Relling MV Clinical pharmacokinetics of paclitaxel Clin Pharmacokinet 1994 27 4 256 69 10.2165/00003088-199427040-00002 7834963

[B17] Haldar S Jena N Croce CM Inactivation of Bcl-2 by phosphorylation Proc Natl Acad Sci USA 1995 92 10 4507 11 PMCID: 41973 10.1073/pnas.92.10.4507 7753834 PMC41973

[B18] Geard CR Jones JM Schiff PB Taxol and radiation J Natl Cancer Inst Monogr 1993 15 89 94 7912535

[B19] Sasaki YF Saga A Akasaka M Yoshida K Nishidate E Su YQ In vivo genotoxicity of ortho-phenylphenol, biphenyl, and thiabendazole detected in multiple mouse organs by the alkaline single cell gel electrophoresis assay Mutat Res 1997 395 2-3 189 98 9465930 10.1016/s1383-5718(97)00168-x

[B20] Nianjun H Cerepnalkoski L Nwankwo JO Dews M Landolph JR Induction of chromosomal aberrations, cytotoxicity, and morphological transformation in mammalian cells by the antiparasitic drug flubendazole and the antineoplastic drug harringtonine Fundam Appl Toxicol 1994 22 2 304 13 10.1006/faat.1994.1034 8005380

[B21] Chu SW Badar S Morris DL Pourgholami MH Potent inhibition of tubulin polymerisation and proliferation of paclitaxel-resistant 1A9PTX22 human ovarian cancer cells by albendazole Anticancer Res 2009 29 10 3791 6 19846910

[B22] Khalilzadeh A Wangoo KT Morris DL Pourgholami MH Epothilone-paclitaxel resistant leukemic cells CEM/dEpoB300 are sensitive to albendazole: Involvement of apoptotic pathways Biochem Pharmacol 2007 74 3 407 14 10.1016/j.bcp.2007.05.006 17560963

